# Weather Classification Using an Automotive LIDAR Sensor Based on Detections on Asphalt and Atmosphere

**DOI:** 10.3390/s20154306

**Published:** 2020-08-01

**Authors:** Jose Roberto Vargas Rivero, Thiemo Gerbich, Valentina Teiluf, Boris Buschardt, Jia Chen

**Affiliations:** 1 Audi AG, Auto-Union-Str., D-85057 Ingolstadt, Germany; jose-roberto.vargas-rivero@audi.de (J.R.V.R.); thiemo.gerbich@audi.de (T.G.); boris.buschardt@audi.de (B.B.); 2 Electrical and Computer Engineering, Technical University of Munich, Theresienstr. 90, D-80333 München, Germany; 3 Department of Electrical, Electronic and Communication Engineering, Friedrich-Alexander University of Erlangen, Schloßplatz 4, D-91054 Erlangen, Germany; valentina.teiluf@fau.de; 4 Institute for Advanced Study, Technical University of Munich, Lichtenbergstraße 2 a, D-85748 Garching, Germany

**Keywords:** autonomous driving, automotive LIDAR, weather conditions, sunlight, classification, atmosphere, street

## Abstract

A semi-/autonomous driving car requires local weather information to identify if it is working inside its operational design domain and adapt itself accordingly. This information can be extracted from changes in the detections of a light detection and ranging (LIDAR) sensor. These changes are caused by modifications in the volumetric scattering of the atmosphere or surface reflection of objects in the field of view of the LIDAR. In order to evaluate the use of an automotive LIDAR as a weather sensor, a LIDAR is placed outdoor in a fixed position for a period of 9 months covering all seasons. As target, an asphalt region from a parking lot is chosen. The collected sensor raw data is labeled depending on the occurring weather conditions as: clear, rain, fog and snow, and the presence of sunlight: with or without background radiation. The influence of different weather types and background radiations on the measurement results is analyzed and different parameters are chosen in order to maximize the classification accuracy. The classification is done per frame in order to provide fast update rates while still keeping an F1 score higher than 80%. Additionally, the field of view is divided into two regions: atmosphere and street, where the influences of different weather types are most notable. The resulting classifiers can be used separately or together increasing the versatility of the system. A possible way of extending the method for a moving platform and alternatives to virtually simulate the scene are also discussed.

## 1. Introduction

One of the main challenges for the safety validation of autonomous driving vehicles lies on the influence of weather phenomena [1,2]. As each of the main sensors, namely LIDAR, radar and cameras increases its sensitivity in order to detect smaller objects faster and hence be able to drive autonomously at higher speeds, the possible influence of environmental perturbations on their perception increases. On the one hand, those perturbations could cause false positives (the term false positives in this paper refers to a false detection in the point cloud before segmentation and classification are done), confuse self-calibration algorithms and reduce the sensor range [3,4]. On the other hand, they could constitute a source of valuable information if the dependencies are known and properly characterized to better evaluate and predict road conditions or adapt its operation mode, for example, [5].

In this paper, we focus on the influence of rain, fog and snow on a LIDAR sensor. Previous results are expanded by considering not only absorption and reflection [2] as well as changes in the reflection characteristics of the target [3], but also the simultaneous influence of a changing ambient illumination in outdoor conditions.

With that objective in mind, a LIDAR was placed outdoors in a static position. As target, an asphalt region of a parking lot was used. The collected point cloud data was separated between detections on the atmosphere and street. The detections on those two areas were analyzed using a classifier in order to identify if, in a static scenario, reliable information about the current weather could be extracted based on the information provided by an automotive LIDAR. Finally, possible applications of the results are shown, including a way of extending the method for use in a moving platform (car/bus/truck) in which sensor and targets move. For the virtual simulation, the use of physically-based rendering is suggested in order to include the effects of a changing background illumination together with changing reflection properties of targets in a reproducible way.

The next section introduces the current state of the art regarding the influence of weather on the LIDAR performance and the use of weather classification algorithms with focus on LIDAR data. Section 3 presents the experimental setup and Section 4 the results for the atmosphere and street regions.

## 2. State of the Art

### 2.1. Influence of Weather on a Performance

One of the main disadvantages of optical sensors (camera and LIDAR) in comparison to radar is their higher performance degradation under the presence of rainfall, fog and snow [6]. In general, the influence of adverse weather on the performance of a LIDAR sensor can be divided as follows:

- Changes in the mechanical, optical and chemical properties of the LIDAR cover like: change in the transmission caused by water absorption [7]. Deformation and change in the refractive index caused by changes in temperature. Changes in its chemical composition due to constant exposure to ultraviolet light [8].
- New layers formed on the LIDAR cover such as: dirt layers [9], water layers (or ice layers) deposited due to the presence of rain, fog, insects and spray from other vehicles.
- Scattering or absorption caused by rain drops, exhaust gasses or other pollutants [10] and dust [11].
- Changes in the optical properties of the target [2] which can be: wet, covered with snow, covered with dirt, etc.
- Changes in the background illumination.

Previous attempts to simulate the impact of weather on the performance of a LIDAR make use of the LIDAR equation [3], which calculates the absorption and scattering coefficients of rain, snow or fog based on the drop size distribution [12]. Given the relationship between drop size distribution and rain intensity, it is possible to use Monte Carlo simulations to calculate a false positives rate caused by the drops [13] for a given rain intensity. Alternatively, the detections can be filtered out using a Kalman filter or dynamic radius outlier removal filters [14,15].

The effect of rain on the reflectance of different targets has also been studied [2,7]. In this case, the absorption and scattering caused by rain is characterized simultaneously with the change in target reflectance by measuring the range, intensity and number of reflected points using a LIDAR sensor for targets placed outdoors. It is found that rain causes a decrement in the intensity and number of detected points for all targets tested, which included materials such as metal, asphalt, concrete and a retro reflective surface, each one placed at a different distance [2]. The authors mention that the change in the reflection characteristics of the target seem to generate an intensity change bigger than the one generated by absorption and scattering caused by rain [2]. The material with the maximum reduction in number of reflected points is found to be asphalt.

This paper will focus on the case in which the LIDAR sensor and target are both placed outdoors. In that way, the effect of a changing background illumination and the change in the optical properties of the LIDARs cover are also included. As target, an asphalt portion of a parking lot is used. Besides physical effects, the influence of sensor internal algorithms that dynamically adapt the performance of the sensor like automatic gain control [16], for example, are left active.

### 2.2. Effect of Water on a Surface’s Transmission and Reflection

For analysis purposes, the effect of water upon the transmission and reflection of a porous material can be divided into three stages: first, the pores of the material are filled with water. When the material reaches saturation, a thin water film forms on top of it. As the water level increases, the characteristics of the material itself lose importance and the reflection starts to depend mostly on the optical properties of water.

The first stage can be understood by considering the change in the optical path length of the material when its pores are filled with water instead of air. This implies a higher probability of absorption of the photons and hence a reduction in its transmission. Regarding reflection, the scattering of the surface changes to a more forward scattering. Consequently, as can also be seen in the visible spectral range, the surface becomes more opaque [7]. A comparison of different dry and wet materials shows an average reflectivity reduction of around 10% for a wavelength of 900 nm and of around 30% for a wavelength of 1.5 µm [1]

The second stage can be analyzed assuming a thin and homogenous water layer. In this case, considering the plastic cover of a LIDAR, for example, a multilayer interface is built consisting of air, plastic or glass, a coating, and a water layer. The Fresnel equations can then be used to calculate the reflectance. In most cases, though, the layer will not be homogeneous. In that case, the surface can be characterized in transmission and reflection by measuring its bidirectional transmittance distribution function (BTDF) and bidirectional reflectance distribution function (BRDF) [17] for the required wavelength.

For further increments in the water level, the reflection can be analyzed by using the bathymetric LIDAR equation [16].

**Figure 1:**
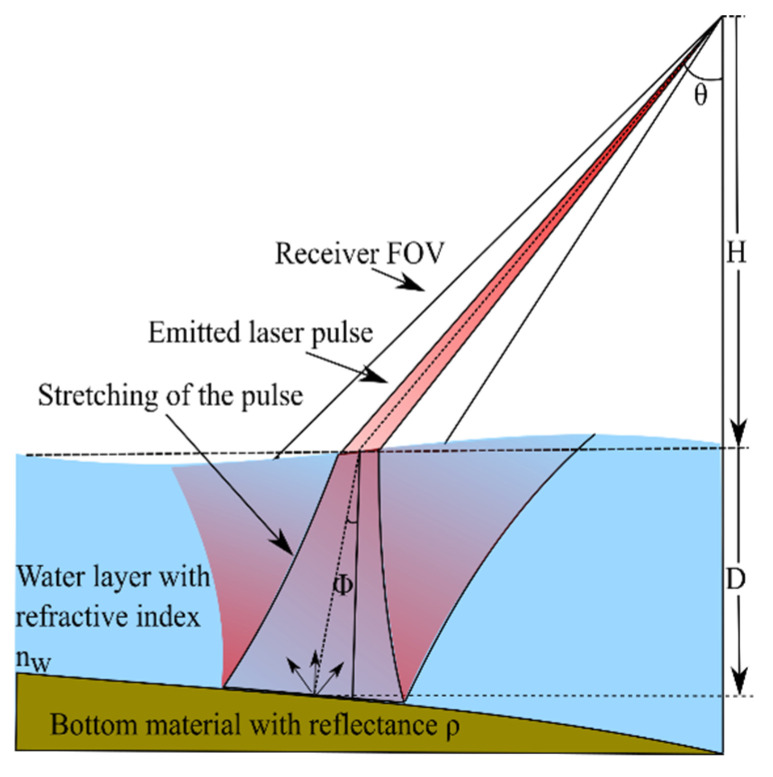
Illustration of the parameters used in the bathymetric LIDAR equation. Although the used LIDAR is not a bathymetric LIDAR the equation is used to show the dependencies present when the amount of water on top of a certain surface is high. These dependencies are used for the analysis of the street region when covered by water in Section 4.

This equation uses the parameters as illustrated in Figure 1. It is defined as:

(1) $$P_{r}=\frac{cP_{T}\rho {cos}^{2}\theta }{{\left(n_{w}H+D\right)}^{2}}\;exp\left(-2\alpha D\sec\phi \right)$$

where $P_{r}$ corresponds to the received power, $P_{T}$ to the transmitted power, ρ to the reflectance of the bottom material, θ to the incident angle of the transmitted laser beam in air, ϕ to the refracted beam angle in water, H to the altitude of the LIDAR above the water, D to the distance between the water surface and the bottom material, $n_{w}$ the refraction index of water, α which combines stretching and attenuation of the pulse, and c is a constant containing sensor-related values [16]. This equation corresponds to the conventional LIDAR range equation for an extended target, with an added exponential decay in a scattering medium corresponding to the Beer-Lambert law.

### 2.3. Effect of Ambient Light on LIDAR Measurements

Since at the end, the information obtained from the LIDAR is based on detections over the noise level, the APD (avalanche photo diode) equation also needs to be considered:

(2) $$SNR=\frac{P_{r}}{N}$$

where $P_{r}$ can be calculated as shown in Equation (1) and N corresponds to the noise which is a combination of the shot noise $N_{S}$, the background noise $N_{B}$, which is proportional to the background light optical power collected by the detector, and the thermal noise $N_{T}$ [1].

(3) $$P\left(N\left(t\right)=n\right)=\frac{1}{\sigma \sqrt{2\pi }}e^{-\frac{1}{2}{\left(\frac{n-\mu }{\sigma }\right)}^{2}}$$

(4) $$I\left(t_{d}\right)=\mu +x\sigma$$

(5) $$TOF=t_{d}-t_{p}$$

**Figure 2:**
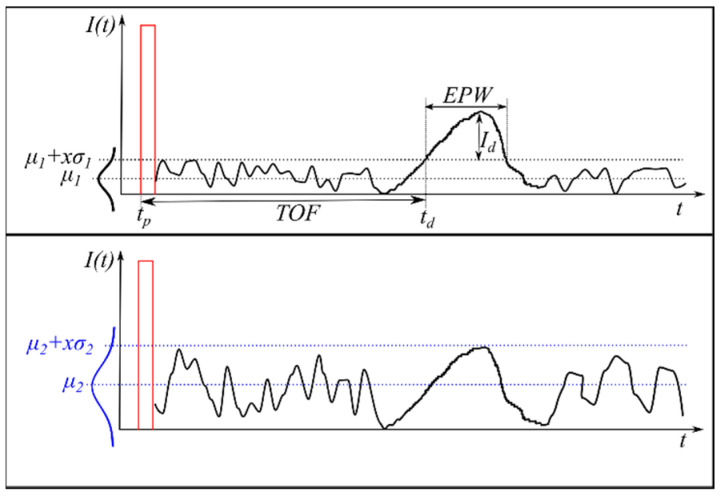
Relation between TOF and intensity for a received pulse. If the noise level increases (blue) no detection is registered. The sent pulse (idealized) is shown in red and the received signal in black.

Finally, the time of flight (TOF) is defined as the time difference between the start of the sent pulse $t_{p}$ and the moment when the detected intensity reaches a certain minimum value $t_{d}$ (5). Assuming a Gaussian distribution for the noise (3), a SNR of 3σ (0.27% probability that a detection is caused by noise) with respect to the mean noise level can be used, for example, in order to define a valid detection (4). If the average noise level increases from $\mu_{1}$ to $\mu_{2}$ as shown in Figure 2, and the shape of the echo pulse remains the same, there is no detection. Correspondingly, the total number of reflected points is reduced. This fact is used for the analysis in the measurement section.

As can be deduced from Figure 2 the intensity of the detection $I_{d}$, its distance, which is calculated using the TOF, and the EPW (Echo pulse width) value also change depending on the noise level. This kind of walk error, which can also be caused by a change in the form of the echo pulse, can in some cases be compensated but not completely avoided [18]. The EPW, which is measured in meters [19], corresponds to the width of the pulse above the noise level. It is proportional to the reflection of the object.

### 2.4. Light Scattering and Absorption by Particles in the Atmosphere

Particles in the atmosphere scatter and absorb the laser light depending on their shape, size and complex index of refraction [20]. Spherical particles with a size parameter α=2πr/λ smaller than 0.1 (Rayleigh regime) tend to have a symmetric forward/backwards scattering. Particles with α values between 0.1 and 50 (Mie regime) have a bigger forward as backward scattering lobe, while particles with α values bigger than 50 (Geometric regime) have a very large forward scattering lobe and almost no backwards scattering [20]. In case of snow, the analysis is more complicated and depends on the exact shape of the crystal, which depends on the temperature [3]. For multiple particles, the scattering coefficient is also proportional to particle concentration and size [21].

Table 1 shows the average radius and size parameter for typical particles in the atmosphere. The size parameter is based on a laser with a wavelength of 905 nm, which is in the range of wavelengths typically used for autonomous driving cars [22,23]. The refractive index for both water and ice at this wavelength has an imaginary part in the order of $10^{-7}$ [24] and hence the single scattering albedo (SSA) for both drops and crystals in the geometric regime approximates 0.53 [25,26]. For fog, it approximates 0.8 [26].

Although the parameters are distributed over a broad range, not all the sizes occur with the same probability, with common sizes for snowflakes around 1 mm [29], for rain drops around 0.2 mm and for fog droplets around 3 µm (Chu Hogg) and 18 µm (Advection) [3].

### 2.5. Weather Classification Using LIDAR

There are different alternatives to evaluate current weather and road friction in the vicinity of a car. One alternative is using the information provided by the vehicle, for example from windshield wipers, fog lights, torque and speed of engine and tires, anti-lock braking system (ABS), electronic stability control (ESC) and traction control system (TCS) intervention events, temperature, global navigation satellite system (GNSS) position, steering wheel angle and breaking signal [30]. Another alternative is to use sensors specific for road surface analysis like polarization cameras or short distance multi-wavelength IR sensors. These sensors use the change in the amount of vertically polarized light or its resonance frequency caused by the different phases of water to classify between ice, snow and mixtures [31]. A third alternative, and the focus of this paper, is to use advanced driver assistance systems (ADAS) sensors like visible spectrum (VIS) cameras, ultrasound, radar or LIDAR whose main purpose is the detection of static and moving objects but whose performance is affected by weather [32]. All these techniques can be used by themselves or combined to provide different levels of classification accuracy [30]. Additionally, the information provided by other cars or sensors can be included using vehicle-to-everything (V2X) technologies [33].

LIDAR sensors have been used to classify aerosols on the atmosphere using the difference between the extinction cross section and backscatter cross section caused by the different types of aerosols. The differences in the linear depolarization ratio and the frequency differences caused by inelastic scattering (Raman LIDAR systems) are also used [34,35]. These kinds of systems are able to provide the type, size and concentration of the different aerosols. A drawback regarding automotive LIDAR systems is that they usually use monochromatic unpolarized light and measure only elastic scattering effects. This is compensated in some systems by providing multi target detection (multiple echoes), which facilitates the differentiation of detections caused by rain, fog or snow from those caused by a solid objects [36,37].

Regarding the types of classifiers used, in the context of removal of detections caused by fog [38], support vector machine (SVM) and K-nearest neighbor (KNN) were used reporting a classification accuracy of heavy fog vs. solid objects higher than 90% (F-Score) with SVM and 79.4% (F-Score) with KNN. The room in which the experiment took place had a size of 5 m by 4 m. A recent study [32] with a focus similar to the present paper used a Velodyne LIDAR puck (VLP) and a SCALA sensor to classify between clear, rain, and fog. They reported a true positive rate higher than 95% for all three classes using the VLP sensor. The SCALA sensor had a true positive rate (TPR) of 99.78% for fog, but it fell to 84.92% for rain and 83.19% for clear using SVM. It is important to mention that the measurements were done in a climate chamber where the visibility values for the fog and rain intensity were kept within relatively constant ranges. The measurements are reported in a region with a size of 10 m by 25 m. A second set of measurements was performed on the road using only the VLP sensor. In this case, only two classes were used: clear and rain. A TPR of 92.45% for rain and 97.60% for clear is reported using KNN. To train the classifier, a parameter vector based on the position of the detection in Cartesian and spherical coordinates as well as the echo number and the intensity (for the VLP sensor) or echo-pulse width (for the SCALA sensor) is used.

In a recent paper [39], the measurement distance was reduced to a region close to the LIDAR cover (<1.6 m). In this case, the classes used were: clean, salt, dirt type 1, dirty type 2 and frost. Images where constructed from two different views: front view image, with layer number on the y axis, horizontal angle on the x axis and echo-pulse width as color and top view image with vertical angle on the y axis radial distance in the x axis and layer number as color. These two image types were used to train a deep neural network reaching a classification accuracy of 77.98%. If only classifying between clean, salt and frost, the accuracy increased up to 95.41%. Although in this case the focus is not weather classification, the state of the LIDAR cover provides an important hint about it.

## 3. Experiment

As mentioned in the introduction, the sensor was placed outdoors and the recorded data was extracted for two regions: atmosphere region (distance from sensor <5 m) and street region (distance from sensor between 33 m and 37 m).

**Figure 3:**
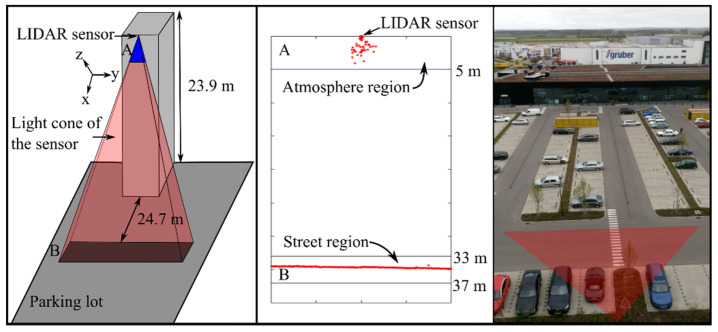
Depiction of the measurement setup. Left: schematic front view of the LIDAR sensor position and its effective field of view (A: atmosphere region, B: street region), the used axis corresponds to the LIDAR coordinate system. Middle: example of the obtained point cloud (birds eye view). Right: Picture from top of the building. The field of view of the sensor is illustrated by the red triangle.

The measurement setup consisted of a LIDAR sensor, which was installed on the roof of a building, as illustrated on the left side of Figure 3 (see Appendix A for detailed setup information). The reflectance of water for non-polarized light coming from air is almost constant and close to 2% up to an angle of around 45° to the surface normal, and increases exponentially [40] above this angle. Hence, an angle close to 45° or less would be convenient in order to preserve a dependency on ρ (see (1)) in the results for thin water layers. In our test setup, an angle of 46° is used.

The recorded data was obtained by activating and deactivating the laser scanner at predefined times, from April to December 2017. In total, 639 h were recorded, during which it was raining 74 h (12%), foggy 45 h (7%) and snowing 9 h (1%).

For the evaluation, the recorded hours were discriminated in four weather classes: Clear, Rain, Fog and Snow. The recorded data was classified using information from a weather station from the Bavarian Environmental Agency [41]. As fog is not reported, we performed the fog measurements manually started and stopped them. The precipitation values are reported with an interval of five minutes and for snow and background radiation each hour (see Appendix B for detailed weather information). The weather station was located at a distance of 7 km from the measurement setup.

Due to an automatic gain control system, which will be further explained in the Results section, only two values were used for background radiation: (1) with background radiation and (2) without background radiation. For each sample, the following procedure was followed:

- It was verified that no people, cars or other objects were passing by during the measurement.
- For Fog, Rain and Snow samples it was verified that the weather remained the same during a period of at least 5 min before and after.
- Clear samples were taken from days were the weather station did not report rain or snow and no fog was seen.
- The samples were randomly chosen over different days in order to increase the variability.

From each sample, which had a duration of 10s, the single frames were extracted. This resulted in a total of 6130 frames without background radiation and 1930 with background radiation for each weather type. The reason for having a lower number of frames during the day is the difficulty of recording snow data on the street region during that time. The total number of frames is nevertheless within the range used for similar classifiers [32,39].

Each frame contains possibly up to thousands of detections distributed on the cover of the sensor, the atmosphere and the street region. Section 4.1 explains the features used for the parameter vector upon which the classification takes place. These features are based on the analysis of each region:

- Region A (Section 4.1.1) corresponds to changes in the optical characteristics of the atmosphere and the LIDAR cover.
- Region B (Section 4.1.2) corresponds to changes in the reflection of the street. The term region is used instead of surface or plane because it includes reflections coming from water drops splash which may be a few cm away from the street surface or also from a snow cover.

In both figures, the distances are measured horizontally across the axis of symmetry of the sensor (x-direction; Cartesian coordinates were used instead of polar coordinates to facilitate the extraction of the detections corresponding to the street region).

## 4. Results

In the first step, the influence of a changing background radiation was investigated. Background radiation is measured in watts per square meters and gives a numeric value for the brightness. During the night, its value is zero, the biggest value measured during the day and also in the group of selected samples was 779 $W/m^{2}$.

The number of scan points shown in Table 2 corresponds to the average of 3800 frames during clear weather. The frames are separated based on their echo number: $n_{e1}$ for the first echo and $n_{e2}$ for the second. It can be seen that with no background radiation, the mean number of scan points is on average 35% higher for the first echo and for the second echo it is 2.3 times higher. This is caused by the reduction on the noise level (as explained in Figure 2) which also causes the increment of the EPW (~30%). When comparing the number of detections in the atmosphere region with and without background radiation (Section 4.1.1) a reduction in the number of detections can also be seen.

### 4.1. Characterization of the Distributions in the Atmosphere and Street Region

**Figure 4:**
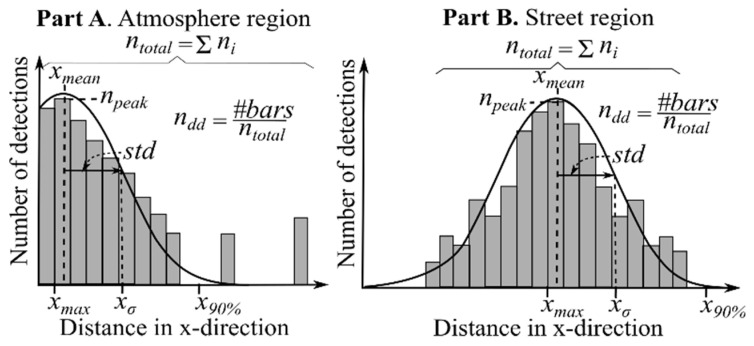
Parameters used to characterize the distributions in the atmosphere and street region.

In order to characterize each of the obtained distributions, a group of seven different parameters were used as shown in Figure 4. The same parameters apply to the atmosphere and street regions with and without background radiation, as in each case they capture the main changes in the shape of the different distributions.

The meaning of each parameter is as follows:

(1) The number of detections in the maximum of the histogram ($n_{peak}$).
(2) The position of the maximum of the histogram in meters ($x_{max}$).
(3) Total number of detections in the region ($n_{total}$).
(4) Mean detection distance in x-direction ($x_{mean}$).
(5) Standard deviation of the detection distance in x-direction ($x_{\sigma }$).
(6) Distance at which 90% of the total number of detections in the region is reached. The points are accumulated per bin from left to right starting at 0 m or 33 m, respectively ($x_{90\%}$).
(7) Number of discrete distances ($n_{dd}$) where detections take place relative to the total number of detections. Especially useful when there is background radiation. This parameter is proportional to the number of bars in the histogram.
(8) Number of first echo detections ($n_{e1}$).
(9) Number of second echo detections ($n_{e2}$).
(10) Number of third echo detections ($n_{e3}$).
(11) Mean value of the echo-pulse width ($EPW_{mean}$).
(12) Standard deviation of the echo-pulse width ($EPW_{std}$).

As will be examined in the discussion section, not all parameters are equally relevant for both regions. For this reason, Table 3 and Table 4 summarize the most relevant parameters for each. Furthermore, they constitute the bases for the presented analysis, which tries to relate the theoretical background about the variation in the optical properties of the LIDAR cover, the street and the atmosphere as presented in Section 2 with the change in the distribution of the detections.

#### 4.1.1. Atmosphere Region

In order to facilitate the interpretation of the data, the results are shown using a histogram with a bin size of 5 cm. For each class, 1900 frames are used.

**Figure 5:**
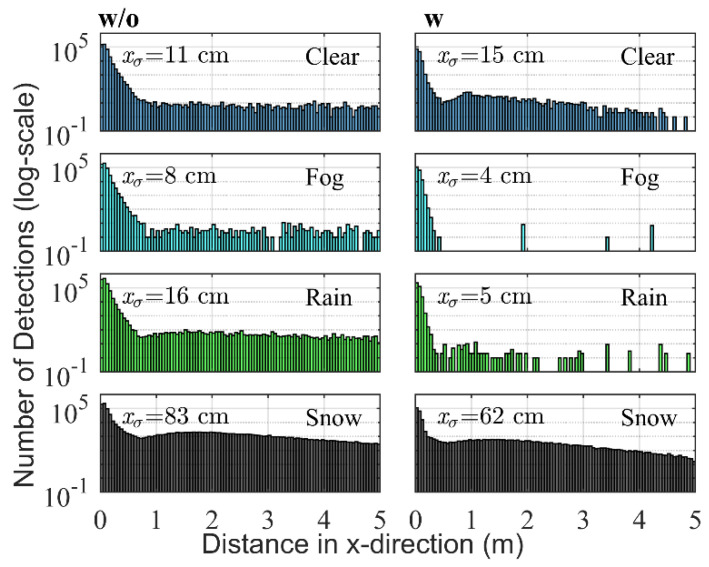
Distribution of the detections for each weather class (1900 frames each) in the atmosphere region (0-5 m) (w/o) without and (w) with background radiation. With $x_{\sigma }$: standard deviation of the detection distance in x-direction in cm.

Figure 5 shows the detections in the atmosphere region without and with background radiation. A logarithmic scale is chosen to point out the differences between the different weather types. The relevant numerical parameters extracted from each distribution are presented in Table 3.

The reflections in the region from 0 to 0.5 m are caused by a combination of multipath reflections on the LIDAR cover and detections on drops or snowflakes. The presence of water or dirt on the cover caused by the different weather types also influences the form of the distribution.

Regarding the region from 0.5 m to 5 m for Clear, some detections are seen. An explanation is that in this case the noise level is at its minimum and hence the sensibility is high. Therefore, some of these detections could have been caused by other particles in the air like pollen, dust or insects.

For Snow, the total number of detections is larger than for Fog, but smaller than for Rain. In comparison with raindrops, the snowflakes tend to be bigger. However, their concentration tends to be smaller (Table 1) and given that the SSAs are similar, it is reasonable to assume that the number of detections is lower. In contrast to Clear and Fog, the distribution for Snow is not homogeneous and shows a maximum around 1.8 m (also slightly visible in the rain distribution w/o), which most probably depends on the optical design of the sensor.

In the case of Fog, it is interesting to see that the total number of detections is lower than for Rain. Fog drops tend to have a higher SSA than snow and raindrops and since they have a bigger backscattering lobe, their concentration is also higher. On the other hand, their size is much smaller which seems to cause - together with the increment in the noise level - that more detections fall under the minimum required voltage (Figure 2) in comparison with raindrops or snowflakes. The increased noise level also explains why the $x_{\sigma }$ value is the lowest of all classes.

As already mentioned with background radiation, the number of detections generally smaller (Table 2) and therefore the differences between the distributions for each weather type (Figure 5 w, compared to Figure 5 w/o) are reduced. This influences the classification accuracy as will be seen in Table 5. Besides that, most of the characteristics already described remain valid. It is interesting to see that even with a higher noise level due to sunlight, the number of detections while snowing maintains a value over distance higher than the other classes. As a result of this, the value of $x_{\sigma }$ is much higher. This effect is caused by the bigger size, on average, of the snowflakes.

#### 4.1.2. Street Region

**Figure 6:**
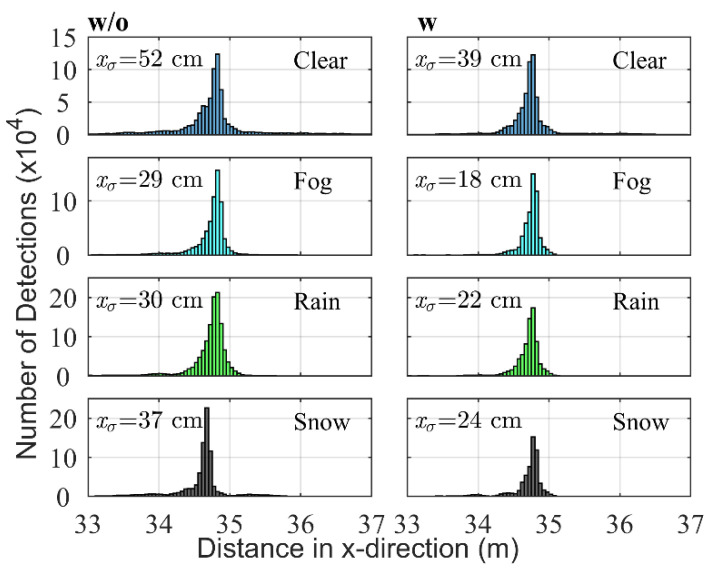
Distribution of the detections for each weather class (1900 frames each) in the street region: (w/o) without and (w) with background radiation. With $x_{\sigma }$: standard deviation of the detection distance in x-direction in cm.

Measurements of the optical properties of the street provide direct information about road friction, which are relevant for self-driving cars to identify situations like aquaplaning. As proof of concept, it is hence interesting to know if the same method can be used for this region. In comparison with the atmosphere, measurements in the street region have a higher degree of uncertainty. Especially during the day and during winter, the use of de-icing or cleaning agents as well as the change in surface temperature could influence measurements. Other factors are the presence of dew, ice and the inhomogeneous distribution of water on the street surface. Nevertheless, the distributions for each weather type (Figure 6) are distinct enough to allow for a useful interpretation and classification. As in Figure 5, the bin size is 5 cm.

Figure 6 w/o shows the detections on the street without background radiation and Figure 6 w with background radiation. In this case, a linear scale is preferable. As for the atmosphere region, the relevant numerical parameters extracted from each distribution are presented in Table 4. However, $n_{total}$ is not present and its place is taken by $x_{mean}$, additionally $n_{e1}$ is replaced by $n_{e2}$ the reason being that for the street region, the new parameters allow for a better separation between the classes as will be analyzed in the Discussion section (feature ablation study).

Considering Figure 6 w/o as well as the information provided by Table 4 first, the EPW (the analysis is based on the mean value of each variable) value is lower as would be expected from the reflection of the different surfaces going from Snow to Clear to Rain. This is reasonable given that the street had a snow cover of a few cm [41], which has a higher albedo (0.8) [42] than dry asphalt (0.12) [43] or wet asphalt (0.03 at 46°). Fog has the lowest EPW value, which contrasts with the results reported using a climate chamber [32] in which the EPW increased proportionally with fog density beyond the values for Rain and Clear. We believe this may apply to surfaces perpendicular to the laser beam in which the backscattering caused by fog particles and the reflection of the surface have the same main direction; in our case, due to the angle of 46° between the laser beam and the surface normal, the net effect is a reduction of the EPW. Besides having the highest EPW, Snow is also characterized by having the smallest $x_{mean}$ value due to the presence of a snow cover.

The class Clear has a $x_{\sigma }$ and $x_{mean}$ value higher than all other classes. This is caused by the more Lambertian reflection lobe of dry asphalt. The presence of humidity or water—as is the case for Fog and Rain—reduces this lobe and increases the forward reflection, as was discussed in Section 2.2. Clear is also characterized by a low number of second echo detections ($n_{e2}$) in comparison with Rain and Snow where the laser beam hits many drops or snowflakes first before hitting the street surface.

Rain is characterized by a relatively low EPW. The number of second echo detections is the highest in all the classes. This, as already mentioned for the atmosphere region, is caused by the higher concentration of drops over snowflakes and their bigger size in comparison with fog droplets. More first echo detections on the drops cause more second echo detections on the street.

Fog has the lowest $x_{\sigma }$ value as happened for the atmosphere region. As mentioned before, this hints to an increment of the average noise level which causes weak detections to vanish (Figure 2). The number of second echo detections is similar to Clear.

Considering Figure 6 w, the EPW for Snow is reduced to a value similar to that of Fog and Rain. This could have been caused by melting of part of the snow cover and the presence of footsteps and wheel marks, which reduced the average albedo of the surface.

Clear has the highest $x_{\sigma }$ and Fog the smallest, as happens without background radiation. Regarding $x_{mean}$ it remains the highest for Clear but now Fog has the second highest value. Those for Snow are similar. This also indicates a partial melting of the snow cover and the reduction of any extra humidity caused by fog on the street surface.

Rain and Snow become very similar with Rain having a higher $n_{peak}$ value. The value of $n_{peak}$ increases when $n_{e2}$ increases and therefore is higher for Snow and Rain and decreases with $x_{\sigma }$. This causes that the $n_{peak}$ values for Clear and Fog with background radiation are higher than without. In general, though, an increase in the noise level due to sunlight causes in both atmosphere and street regions a reduction of the mean values of all the parameters. The next section presents the classification results and relates them with the analysis presented in this section.

### 4.2. Classifier

The results show that there is enough variability in the data for classification. However, a single sample by itself cannot be accurately classified, especially with background radiation when the number of detections is low. For that reason, all frames are used to train a classification algorithm. The inputs for the classifier are the parameters shown in Figure 4, the most important of which were discussed in the previous section: number of detections in the maximum of the histogram ($n_{peak}$), mean detection distance in x-direction ($x_{mean}$), standard deviation of the detection distance in x-direction ($x_{\sigma }$), number of second echo detections ($n_{e2}$) and mean value of the echo-pulse width ($EPW_{mean}$).

For the classification, KNN was employed due to its good performance and use in previous studies [32,38]. To balance the number of samples per class random under sampling was used. With the aim of allowing for comparison and avoiding bias, the same classifier was used in all cases. Five-fold cross validation was used; meaning that four fifths of all data is used for training and one fifth for validation. The process is repeated five times with different partitions of the data, the result is then averaged. The confusion matrix shows the number of samples which where correctly classified in its diagonal. A gray scale is used to facilitate the interpretation going from white for zero frames to black for the maximum number of frames (6130 without and 1930 with background radiation). The F-score is presented as metric. Being the harmonic mean of the precision and recall metrics [44], it provides a good evaluation of the classifier’s performance.

#### 4.2.1. Atmosphere Region

As shown in Table 5, the F-Score for Snow and Rain for the atmosphere region is higher than 94% in both cases. Without background radiation, it is higher than 99%. This is due to the characteristically high $x_{\sigma }$ value of Snow and the high amount of total detections $n_{total}$ and first echo detection $n_{e1}$ of Rain in comparison with the other two classes.

Clear and Fog have a higher F-Score with and without background radiation. The reduction of the average $x_{\sigma }$ values for Fog during the day to values smaller than those for Clear reduces the number of misclassifications between the two classes. This, on the other hand, has as disadvantage that more frames are incorrectly classified between Rain and Fog as the similarities between the two classes increase.

#### 4.2.2. Street Region

The street region (Table 6) shares some similarities with the atmosphere region. As before, Snow and Rain have the highest classification values. The values for Clear and Fog are slightly better, as fewer frames are confused between the two classes. This happens mostly because the variations between the $x_{\sigma }$ values are higher. Misclassifications between Rain and Fog are also far less common. This coincides with previous results [2] in which it is mentioned that changes in surface reflection are the ones that cause the most notable effects on the detections.

## 5. Discussion

In this section, the obtained results are compared with known weather classification results using LIDAR. Furthermore, feature ablation is used to reduce the number of features to the most relevant ones while simultaneously increasing in some classes the classification accuracy. Finally, the possibility of using the same classifiers in a moving platform like a car or truck, for example, is briefly discussed as well as ways to improve accuracy and robustness. The section finishes with a short note about simulation.

### 5.1. Atmosphere Region

Compared with previous results mentioned in Section 2.5, the classifier for Fog has a slightly lower F-Score: 84.4 (w/o BR (Background radiation)) and 89.8 (w BR) than a classifier trained specifically to distinguish between fog an solid objects (F-Score: 90.1) [38]. When compared to the results obtained using a climate chamber, our classifier has a TPR for Fog of 83.7% (w/o BR) and 92.5% (w BR) versus 99.8% [32]. The slightly lower results are mostly a consequence of the innate variability of the outdoor measurement as the parameters and algorithm used for the classification are similar. In case of Rain and Clear, this variability could have been an advantage as in both cases the TPRs are higher. For Rain, our classifier has a TPR of 98.8% (w/o BR) and 97.2% (w BR) compared to 84.9% in a climate chamber [32]. For Clear, the values are 85.0% (w/o BR) and 91.4% (w BR) compared to 83.2% in a climate chamber [32].

### 5.2. Street Region

For this region, the results are better. Consequently, only the TPR for Fog is lower when comparing it with the result obtained in a climate chamber [32]: 94.8% (w/o BR) and 98.4% (w BR) versus 99.8%. In all other cases, our classifier provides better results.

### 5.3. Feature Ablation Study

Some features provide a very small separation between the classes and in some cases may even reduce the classification accuracy. For that reason, a feature ablation study is done. The results are presented in Table 7 for the atmosphere region and Table 8 for the street region. In each case, the classifier is trained with all features minus one, the results are compared with the original result using all features. The F1-score is used as evaluation parameter as was done in Table 5 and Table 6.

Half of the features seem to have a very small impact on the classification. The number of discrete distances ($n_{dd}$) when removed mostly improves the classification results while, for example, removing the mean value of the EPW $\left(EPW_{mean}\right)$ notably reduces the classification values. Something similar happens for the street region. In this case, besides the ($n_{dd}$) the standard deviation of the EPW ($EPW_{std}$) also seems to have a detrimental impact on the classification.

Based on these results the number of parameters used for the classification was reduced with minor reduction in the F1-Scores and in half of the cases with minor increases. The final classification results are presented in Table 9 and Table 10.

While for the atmosphere region: Clear, Fog and Snow (w BR) improve, all classes with background radiation improve for the street region.

### 5.4. Extension for a Moving Platform (Street)

In a dynamic environment, the surface of the road doesn’t remain constant with respect to the sensor, additionally other objects block dynamically the field of view of the sensor, the road itself changes and the effect of the wind becomes more important. Nevertheless, it should be possible to use a ground plane classification algorithm to select a region on the road as long as it is not blocked. If the measurement is done for sufficient time in order to compensate for the surface deviations, a similar analysis as done for the street before could be used. The change in the reflection of the road would also need to be considered.

### 5.5. Increasing Classification Accuracy and Robustness

Much more snow data needs to be collected with as many different types of snow as possible. Specifically for the street, the measurement should be repeated using different types of asphalt; additionally, a direct measurement of the state of the surface needs to be done using a reference sensor in order to reduce the uncertainty of the measurements. There are already special sensors that can accomplish this task, but they are in most cases not designed for automotive applications. This, together with the advantage of using a sensor, which is already installed in various car models, are the main motivations for further research.

With enough data it may be possible to return a probability per class instead of a binary classification, this may increase the robustness and usefulness of the method allowing for weather combinations. Furthermore, the acquisition of a weather station which can be placed next to the sensor would increase accuracy.

Finally, the use of the same method with other LIDAR sensors would provide important information about the influence of internal sensor calibration algorithms and architecture upon the most important classification parameters and the best size for the atmosphere region.

### 5.6. Virtual Simulation

Some form of ray tracing is usually used for the simulation of LIDAR sensors [45,46]. Regarding the atmosphere, it is possible to use volumetric scattering or particle systems. Particles would have the advantage of being able to interact with forces. For example, the effect of wind could be simulated, but the computational cost would be higher. The change in the background illumination can be simulated using a lamp placed at infinity (parallel rays without distance falloff). For the street, different BRDFs and textures can be used to simulate the reflection properties and roughness.

## 6. Conclusions

This paper shows that it is possible to use an automotive LIDAR sensor to differentiate between four different weather types: Clear, Rain, Fog and Snow. Two alternatives were presented, using the detections in the atmosphere region or using the detections on asphalt. Additionally, the presence of background radiation was taken into consideration. For the atmosphere region the F1-Scores were: Clear (85.30 w/o BR, 92.54 w BR), Rain (98.82 w/o BR, 93.27 w BR), Fog (84.82 w/o BR, 89.01 w BR) and Snow (99.27 w/o BR, 96.86 w BR). For the street region the F1-Scores were: Clear (91.29 w/o BR, 96.67 w BR), Rain (99.43 w/o BR, 99.09 w BR), Fog (91.42 w/o BR, 96.81 w BR) and snow (99.76 w/o BR, 98.86 w BR)

Different parameters were defined and used as input for the classifier based on the changes in the distribution of the detection distances for each weather type as well as the number of echoes and the EPW.

For the atmosphere region, the average particle size and density seem to be the most important physical parameters that influences the total number of detections. For the street region, the surface albedo and scattering profile are the most important parameters.

The presence of background illumination increases the noise and hence reduces the total number of detections and the EPW.

The classification based on the atmospheric region does not depend on the road surface or the angle of the LIDAR sensor and hence can be directly used in a moving car. The classification results can be combined with the information provided by other sensors in the car or cooperative with other cars [47] in order to increase the confidence level.

An algorithm like the one presented can help to improve the evaluation of road friction and be an input for other sensors or semi-/autonomous functions whose performance depends on local weather [48,49].

## Figures and Tables

**Table 1:** Typical atmospheric scattering particles with their average size parameter for a wavelength from 905 nm and concentration [21,24,27,28,29].

Type	Radius (µm)	Size Parameter	Concentration
Air molecules	0.0001	0.0007	<3 × $10^{25}\;m^{-3}$
Haze, smoke, dust	0.01–1	0.07	$10^{5}$–5 × $10^{10}\;m^{-3}$
Fog	1–20	7–139	$10^{6}$–5 × $10^{9\;}m^{-3}$
Rain	100–10000	694–69427	10–$10^{3}$ $m^{-3}$
Snow	1000–5000	6943–34714	$10^{-2}$–$10^{3}\;m^{-3}$
Hail	5000–50000	34714–347137	$10^{-2}$–1 $m^{-3}$

**Table 2:** Influence of background radiation on the total number of echoes and EPW for 1900 frames during the day and 1900 during the night including both regions during clear weather. The results show mean value ± standard deviation.

$W/m^{2}$	$n_{e1}$	$n_{e2}$	EPW [m]
Global radiation=0	257 ± 19	66 ± 67	1.21 ± 0.39
Global radiation>0	190 ± 110	20 ± 25	0.93 ± 0.27

**Table 3:** Most important parameters for the atmosphere region (0–5 m)*. (w/o) Without background radiation, (w) with background radiation. With $n_{total}$: total number of detections in the region, $n_{peak}$: number of detections in the maximum, $x_{\sigma }$: standard deviation of the detection distance, $n_{e1}$: number of first echo detections and EPW: echo-pulse width. The results show mean value ± standard deviation.

	Clear		Fog		Rain		Snow	
	w	w/o	w	w/o	w	w/o	w	w/o
$n_{total}$	86 ± 34	244 ± 16	114 ± 14	313 ± 55	238 ± 161	661 ± 113	137 ± 13	344 ± 57
$n_{peak}$	55 ± 18	113 ± 19	74 ± 14	124 ± 34	159 ± 124	287 ± 60	79 ± 14	126 ± 21
$x_{\sigma }$ [cm]	5 ± 11	10 ± 8	3 ± 2	7 ± 5	3 ± 3	15 ± 8	57 ± 22	84 ± 23
$n_{e1}$	86 ± 34	244 ± 16	114 ± 14	313 ± 55	238 ± 161	659 ± 112	135 ± 12	324 ± 46
EPW [m]	0.75 ± 0.29	0.95 ± 0.35	0.82 ± 0.29	0.94 ± 0.34	0.86 ± 0.30	1.00 ± 0.35	0.86 ± 0.32	1.02 ± 0.38

* In contrast to Figure 5 and Figure 6, in which multiple frames are used and the parameters are calculated once for all of them, in this table as well as in Table 4 the parameters are calculated per frame and compared.

**Table 4:** Most important parameters for the street region (33–37 m). (w/o) Without background radiation, (w) with background radiation. With $n_{peak}$: number of detections in the maximum, $x_{mean}$: mean detection distance, $x_{\sigma }$: standard deviation of the detection distance, $n_{e2}$: number of second echo detections and EPW: echo-pulse width. The results show mean value ± standard deviation.

	Clear		Fog		Rain		Snow	
	w	w/o	w	w/o	w	w/o	w	w/o
$n_{peak}$	77 ± 39	45 ± 37	88 ± 30	72 ± 26	97 ± 39	125 ± 33	89 ± 30	132 ± 33
$x_{mean}$ [cm]*	74 ± 15	96 ± 27	73 ± 02	74 ± 03	69 ± 03	71 ± 03	70 ± 05	60 ± 03
$x_{\sigma }$ [cm]	27 ± 22	68 ± 30	17 ± 5	24 ± 5	21 ± 5	31 ± 7	22 ± 8	37 ± 9
$n_{e2}$	40 ± 21	131 ± 20	37 ± 11	128 ± 21	52 ± 19	300 ± 52	51 ± 9	193 ± 32
EPW [m]	1.10 ± 0.24	1.48 ± 0.23	1.02 ± 0.25	1.09 ± 0.30	1.01 ± 0.26	1.22 ± 0.33	1.03 ± 0.26	1.63 ± 0.30

* This value is shown after subtracting the same value (34 m) from each class in order to show the differences in cm.

**Table 5:** Confusion matrix for the atmosphere region without background radiation (left, 6130 samples per class) and with background radiation (right, 1930 samples per class) (classifier: Weighted KNN).

		Predicted Class							
		Without Background Radiation				With Background Radiation			
		Clear	Rain	Fog	Snow	Clear	Rain	Fog	Snow
Actual Class	Clear	5284	1	844	1	1670	32	188	40
	Rain	3	6055	32	40	0	1877	43	10
	Fog	980	16	5132	2	58	80	1785	7
	Snow	2	19	1	6108	0	50	42	1838
	F-Score	84.97	99.04	84.36	99.42	91.42	94.74	89.78	96.23

**Table 6:** Confusion matrix for the street region without background radiation (left, 6130 samples per class) and with background radiation (right, 1930 samples per class) (classifier: Weighted KNN).

		Predicted Class							
		Without Background Radiation				With Background Radiation			
		Clear	Rain	Fog	Snow	Clear	Rain	Fog	Snow
Actual Class	Clear	5462	0	667	1	1761	32	112	25
	Rain	0	6078	52	0	5	1912	1	12
	Fog	315	6	5809	0	13	0	1900	17
	Snow	11	0	3	6116	0	0	13	1917
	F-Score	91.62	99.45	91.67	99.89	95.04	98.71	96.11	98.31

**Table 7:** Results of the feature ablation analysis for the atmosphere region using the F1-Score as evaluation parameter. In comparison with the score using all features, the features are marked with ✓ when the F1-Score for two or more classes decreases more than 0.4 (marked in grey), with ✕ when increases more than 0.4 (marked in blue) and with **-** in other cases (All-$EPW_{std}$ is marked as neutral because the F1-Score of Clear increases).

Feature	Atmosphere w/o BR (F1-Score)				Atmosphere w BR (F1-Score)				Evaluation
	Clear	Rain	Fog	Snow	Clear	Rain	Fog	Snow	
**All**	84.97	99.04	84.36	99.42	91.42	94.74	89.78	96.23	
All-$n_{peak}$	85.11	98.94	84.48	99.36	91.10	94.23	89.15	96.05	✓
All-$x_{max}$	84.83	99.11	84.34	99.41	91.54	94.76	89.65	96.02	-
All-$n_{total}$	84.50	99.04	83.85	99.40	91.04	94.65	89.16	96.10	✓
All-$x_{mean}$	84.94	99.11	84.24	99.48	91.60	94.54	89.74	96.11	-
All-$x_{\sigma }$	84.71	99.02	84.01	99.31	91.40	94.38	89.12	95.17	✓
All-$x_{90\%}$	84.76	99.09	84.18	99.47	91.36	94.66	89.49	96.16	-
All-$n_{dd}$	85.62	99.12	85.20	99.38	91.25	94.67	89.64	96.13	✕
All-$n_{e1}$	84.43	99.04	83.84	99.42	91.20	94.86	89.26	96.18	✓
All-$n_{e2}$	85.21	99.09	84.50	99.49	91.37	94.43	89.55	96.02	-
All-$n_{e3}$	85.25	99.11	84.63	99.44	91.50	94.59	89.63	96.21	-
All-$EPW_{mean}$	82.01	98.09	80.71	98.79	91.55	92.51	88.22	95.48	✓
All-$EPW_{std}$	84.86	98.92	84.21	99.47	91.93	93.59	88.55	96.42	-

**Table 8:** Results of the feature ablation analysis for the atmosphere region using the F1-Score as evaluation parameter. In comparison with the score using all features the features are marked with ✓ when the F1-Score for two or more classes decreases more than 0.4 (marked in gray), with ✕ when increases more than 0.4 (marked in blue) and with **-** in other cases.

Feature	Street w/o BR (F1-Score)				Street w BR (F1-Score)				Evaluation
	Clear	Rain	Fog	Snow	Clear	Rain	Fog	Snow	
**All**	91.62	99.45	91.67	99.89	95.04	98.71	96.11	98.31	
All-$n_{peak}$	91.19	99.51	91.42	99.81	95.18	98.71	96.23	98.15	✓
All-$x_{max}$	91.62	99.37	91.66	99.85	94.94	98.79	95.89	98.18	-
All-$n_{total}$	91.63	99.44	91.67	99.90	94.89	98.76	95.93	98.23	-
All-$x_{mean}$	91.58	99.23	91.42	99.90	94.09	98.63	94.80	98.10	✓
All-$x_{\sigma }$	91.67	99.50	91.77	99.89	94.70	98.50	95.94	97.87	✓
All-$x_{90\%}$	91.44	99.46	91.58	99.82	94.90	98.66	96.04	98.31	-
All-$n_{dd}$	91.57	99.62	91.75	99.89	95.98	98.91	96.67	98.39	✕
All-$n_{e1}$	91.35	99.37	91.32	99.89	94.76	98.61	95.91	98.05	-
All-$n_{e2}$	91.32	99.42	91.33	99.91	93.76	97.86	95.31	96.51	✓
All-$n_{e3}$	91.55	99.45	91.68	99.77	96.15	98.87	96.95	99.07	-
All-$EPW_{mean}$	91.07	99.27	90.99	99.86	94.00	97.91	95.49	97.67	✓
All-$EPW_{std}$	92.05	99.55	92.08	99.95	95.79	99.07	96.61	98.59	✕

**Table 9:** F1-Score per class for the atmosphere region when using only the parameters: $n_{total},$ $n_{peak,}$ $x_{\sigma },$ $n_{e1},$ $EPW_{mean}$.

Atmosphere w/o BR (F1-Score)				Atmosphere w BR (F1-Score)			
Clear	Rain	Fog	Snow	Clear	Rain	Fog	Snow
85.30	98.82	84.82	99.27	92.54	93.27	89.01	96.86

**Table 10:** F1-Score per class for the street region when using the parameters: $n_{peak}$, $x_{mean}$, $x_{\sigma },$ $n_{e2}$, $EPW_{mean}$.

Street w/o BR (F1-Score)				Street w BR (F1-Score)			
Clear	Rain	Fog	Snow	Clear	Rain	Fog	Snow
91.29	99.43	91.42	99.76	96.67	99.09	96.81	98.86

**Figure A1:**
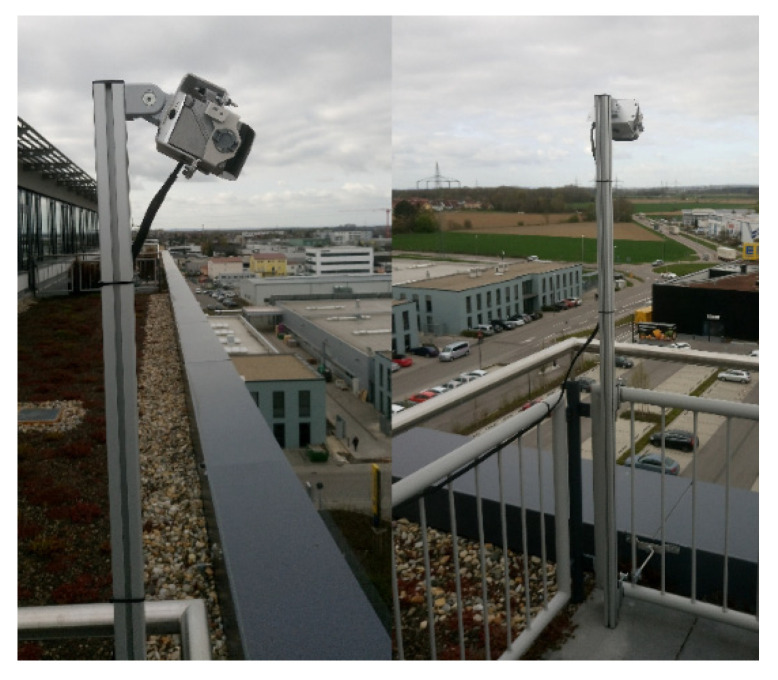
Measurement setup showing the placement of the LIDAR sensor. The location was chosen to avoid the influence of dust caused by cars and local weather variations caused by nearby high buildings or other structures.

**Figure A2:**
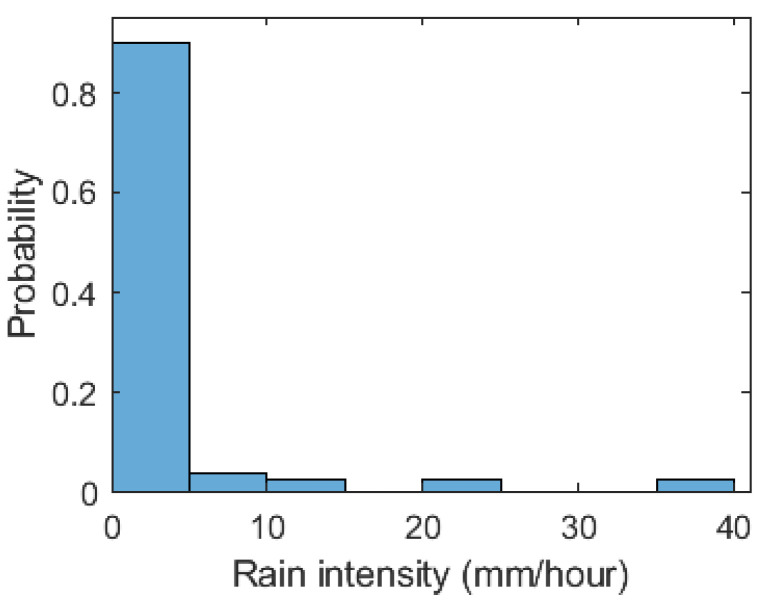
Histogram of the rain intensity for the Rain samples. In most cases the rain intensity was less than 5 mm/h.

**Figure A3:**
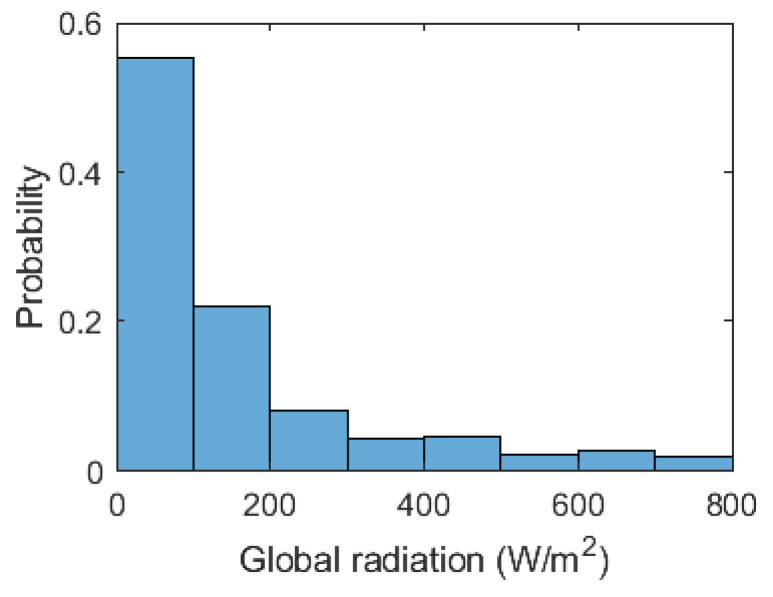
Histogram of the global radiation for all samples with background radiation. The average value varies depending on the class: 343 W/$m^{2}$ (Clear), 104 W/$m^{2}$ (Rain), 86 W/$m^{2}$ (Fog), 13 W/$m^{2}$ (Snow).

## Abbreviations

ABS	Anti-lock braking system
ADAS	Advanced driver assistance systems
BR	Background radiation
EPW	Echo pulse width
ESC	Electronic stability control
GNSS	Global navigation satellite system
KNN	K-nearest neighbor
LIDAR	Light detection and ranging
SVM	Support vector machine
TCS	Traction control system
TPR	True positive rate
V2X	Vehicle-to-everything
VIS	Visible spectrum (340 to 740 nm)
VLP	Velodyne LIDAR puck

## Appendix A

Placement of the LIDAR sensor.

## Appendix B

Relevant probability distributions of weather-related variables.
